# Lymphatic density and metastatic spread in human malignant melanoma

**DOI:** 10.1038/sj.bjc.6601571

**Published:** 2004-02-03

**Authors:** J D Shields, M Borsetti, H Rigby, S J Harper, P S Mortimer, J R Levick, A Orlando, D O Bates

**Affiliations:** 1 Microvascular Research Laboratories, Department of Physiology, University of Bristol, Preclinical Veterinary School, Southwell Street, Bristol BS2 8EJ, UK; 2 Department of Plastic Surgery, Frenchay Hospital, Bristol, UK; 3 Department of Pathology, Frenchay Hospital, Bristol, UK; 4 Department of Physiological Medicine, St George's Hospital Medical School, London; 5 Department of Physiology, St George's Hospital Medical School, London

**Keywords:** malignant melanoma, lymphangiogenesis, LYVE-1, metastasis, VEGF-C

## Abstract

Malignant melanoma (MM), the most common cause of skin cancer deaths, metastasises to regional lymph nodes. In animal models of other cancers, lymphatic growth is associated with metastasis. To assess if lymphatic density (LD) was increased in human MM, and its association with metastasis, we measured LD inside and around archival MM samples (MM, *n*=21), and compared them with normal dermis (*n*=11), basal cell carcinoma (BCC, *n*=6) and Merkel cell carcinoma (MCC), a skin tumour thought to metastasise through a vascular route (MCC, *n*=6). Lymphatic capillary density (mm^−2^), as determined by immunohistochemical staining with the lymphatic specific marker LYVE-1, was significantly increased around MM (10.0±2.5 mm^−2^) compared with normal dermis (2.4±0.9 mm^−2^), BCC (3.0±0.9 mm^−2^) and MCC (2.4±1.4 mm^−2^) (*P*<0.0001). There was a small decrease in LD inside MM (1.1±0.7 mm^−2^) compared with normal dermis, but a highly significant decrease in BCC (0.14±0.13) and MCC (0.12±2.4) (*P*<0.01 Kruskal–Wallis). Astonishingly, LD discriminated between melanomas that subsequently metastasised (12.8±1.6 mm^−2^) and those that did not (5.4±1.1 mm^−2^, *P*<0.01, Mann–Whitney). Lymphatic invasion by tumour cells was seen mainly in MM that metastasised (70% compared with 12% not metastasising, *P*<0.05 Fisher's Exact test). The results show that LD was increased around MMs, and that LD and tumour cell invasion of lymphatics may help to predict metastasis. To this end, a prognostic index was calculated using LD, lymphatic invasion and thickness that clearly discriminated metastatic from nonmetastatic tumours.

Malignant melanoma (MM) results in 1600 deaths each year in the UK, due to metastatic spread of the disease (Cancer Research UK, 2002). The most common site of metastatic disease in melanoma is the regional lymph nodes. Currently, tumour thickness is the most reliable prognostic factor and predictor of recurrence for primary cutaneous melanoma. Melanomas are therefore commonly referred to as *thin* (<1.0 mm thick), *intermediate* (1.0–4.0 mm) and *thick* (>4.0 mm). Overall, 10-year survival probabilities for node-negative patients range from >85% for thin melanomas to ⩽45% for thick melanomas (Stadelmann *et al*, 1998). Metastatic spread to the lymph nodes dramatically reduces the overall survival rate at 10 years to 35% (White *et al*, 2002b) and by the time regional nodal metastases are clinically obvious, 70–85% of patients have other distant metastases. A significant proportion (15%) of patients with thin tumours (<1 mm) go on to develop metastatic disease and there are currently no prognostic indicators for which patients with thin MMs will develop metastasis.

Since most melanomas metastasise to the lymph nodes, it has been postulated for many years that they spread through the lymphatic system (Elias *et al*, 1977). It is not clear, however, whether (a) the malignant cells migrate into the intratumoral lymphatics once the tumour has grown to enclose pre-existing lymphatics; (b) the melanoma cells stimulate lymphatic growth into or around the tumour; or (c) malignant cells migrate out of the tumour mass and invade pre-existing lymphatics in the normal tissue on the periphery of the tumour. Recent work in animal models showed that the incidence of metastasis is increased in tumours expressing endothelial growth factors such as vascular endothelial growth factor C (VEGF-C) (Skobe *et al*, 2001b) and VEGF-D (Stacker *et al*, 2001). Furthermore, VEGF-C expression, driven by the rat insulin promoter, resulted in the development of *β*-cell tumours surrounded by an extensive lymphatic network, in which tumour cell masses were observed. These mice also had an increased rate of lymph node metastasis (Mandriota *et al*, 2001). These studies have led to the hypothesis that tumours that stimulate lymphangiogenesis or angiogenesis will be more likely to metastasise than tumours that do not. This should result in more vessels surrounding tumours that metastasise. Furthermore, several recent reports have suggested that tumour derived expression of VEGF-C and VEGF-D may have prognostic value as indicators of metastatic spread in some prevalent human cancers (White *et al*, 2002a; Kishimoto *et al*, 2003; Nakamura *et al*, 2003a, 2003b; Schietroma *et al*, 2003; Yokoyama *et al*, 2003). The density of blood vessels surrounding melanoma correlates poorly, however, with tumour growth and metastasis (Foss *et al*, 1996; Straume *et al*, 1999), despite the link between VEGF-A expression and tumour progression (Erhard *et al*, 1996). The number of lymphatic vessels associated with MM has not previously been clearly identified. There have been some attempts to do this by differential staining of basement membrane (de Waal *et al*, 1997; Fallowfield and Cook, 1990), but neither of these showed any change in the density of basement membrane-free vessels (assumed to be lymphatics). The lack of lymphatic specific markers, however, has prevented the clear identification of surrounding lymph vessels or in tumours.

We have tested the hypothesis that lymphangiogenesis favours nodal metastasis by comparing three different tumours with differing patterns of metastasis, namely MM, Merkel cell carcinoma (MCC) and basal cell carcinoma (BCC). Since BCC does not metastasise, we hypothesised that LD will be low in and around BCC, whereas it should be high around the readily metastasising MM. Merkel cell carcinoma is a highly aggressive, invasive and usually metastasising tumour that often presents with distant metastasis even in the absence of lymph node involvement. It metastasises to the lymph nodes in approximately half the presenting patients, 69% of whom die within the first 3 years (Medina-Franco *et al*, 2001), but the tendency to metastasise to occult regions suggests that MCC is able to metastasise by a vascular rather than a lymphatic route. We hypothesised therefore that LD will be low in and around MCC as well as BCC.

The inability to distinguish lymphatics from blood vessels has been a limiting factor in the assessment of lymphatic involvement in tumours. Over the last 3 years, an increasing number of lymphatic specific molecules have been identified (Podgrabinska *et al*, 2002). One of the first of these was the lymphatic hyaluronan receptor, LYVE-1 (Banerji *et al*, 1999). This has been shown to be expressed almost exclusively on lymphatic endothelium, and antibodies to LYVE-1 have been used to identify lymphatics in and around normal skin (Banerji *et al*, 1999), cornea (Cursiefen *et al*, 2002) head and neck (Beasley *et al*, 2002) and breast cancers(Mattila *et al*, 2002). LYVE-1 is expressed on lymphatics in all tissues so far investigated, and also on blood liver sinusoids (Mouta Carreira *et al*, 2001). In the skin, however, staining appears to be exclusive to the lymphatic endothelial cells. We have therefore used this novel and specific marker to investigate lymphatic densities in skin tumours.

## METHODS AND MATERIALS

### Materials

Rabbit anti-human LYVE-1 was a generous gift from Dr David Jackson, Oxford, UK; Antibodies to PECAM-1 (sc-1506) VEGFR-3 (sc-321), and VEGF-C (sc-7133) were purchased from Santa Cruz Biotechnology (Santa Cruz, USA), VEGF-D (AF286) from R&D Systems and Ki-67 (NA59) from Oncogene.

### Patient details

Control skin was collected from the breast of six patients undergoing breast reduction surgery (total *n*=11) at Frenchay Hospital, Bristol, with Local Ethics Committee approval (North Bristol NHS Trust). In total, 21 MM samples, six BCC samples and six MCC samples were fixed at the time of excision and embedded in paraffin. All tumour samples were archived tissue stored as paraffin-embedded blocks. There were no significant differences between follow-up times for metastatic and nonmetastatic melanoma (time between excision of the tumour and the analysis of the lymphatic densities) (mean±s.e.m.; 5.7±1.4 years, metastatic, 8.3±1.5 years, nonmetastatic, *P*>0.1 *t*-test). The thicknesses (3.4±0.8 mm metastatic, 2.3±0.6 mm nonmetastatic) were also not significantly different (*P*>0.1, *t*-test).

### Assessment of metastasis

Patients were seen every 3–6 months from the time of excision, depending on the thickness of the melanoma. Of the 21 MM patients, 13 had subsequently developed metastasis diagnosed according to the normal clinical practice, and eight were still free of any form of clinically detectable spread. Only two of the patients had died since the melanoma was originally removed, both from metastasis.

### Immunohistochemistry

Serial sections of paraffin-embedded melanoma samples were cut, dewaxed and rehydrated prior to microwave antigen retrieval (800 w) in tris/EDTA (Trizma, 100 mM; EDTA, 2 mM), pH 9 for 8 min. Endogenous peroxidase activity was blocked by incubation for 5 min in 3% hydrogen peroxide followed by two phosphate-buffered saline (PBS) washes (PBS (mM): NaCl, 137; KCl, 2.68; Na_2_HPO_4_, 10; KH_2_PO_4_, 1.76). Nonspecific binding was prevented by incubation in normal serum for 20 min in a humid chamber (for LYVE-1, 5% human serum w v^−1^ in PBS; for PECAM-1, VEGF-C, VEGF-D and Ki67, 1.5% v v^−1^ horse serum in PBS and for VEGFR-3, 1.5% vv^−1^ goat serum in PBS). Slides were incubated in antibodies to LYVE-1 (4.2 *μ*g ml^−1^), PECAM 1 (8 *μ*g ml^−1^), VEGF-C (1.14 *μ*g ml^−1^), VEGF-D (10 *μ*g ml^−1^), VEGFR-3 (2 *μ*g ml^−1^) or Ki67 (4 *μ*g ml^−1^), or normal rabbit IgG, goat IgG or mouse IgG at the appropriate concentration, overnight at 4°C. The slides were washed twice (PBS/Tween, 0.05% v v^−1^) and the nonimmune block was repeated. The primary antibody was detected with a biotinylated goat anti-rabbit (LYVE-1, VEGFR-3), horse anti-goat (PECAM-1, VEGF-C, VEGF-D) or horse anti-mouse (Ki-67) secondary antibody for 30 min at room temperature. Following two washes, slides were incubated in standard avidin biotin complex (elite ABC kit for VEGF-C) for 30 min at room temperature, washed twice, visualised using DAB, rinsed in distilled water and counterstaining with haematoxylin. Sections were dehydrated and mounted in DPX. The results depend on identification of the lymphatic specific marker, LYVE-1. Although expressed on liver sinusoids (Mouta Carreira *et al*, 2001), LYVE-1 was found to be lymphatic specific in skin and preferential to other markers of lymphatics such as VEGFR-3. LYVE-1, rather than VEGFR-3, was used to count lymphatics because, although VEGFR-3 was clearly expressed on lymphatic endothelial cells, it was also detected on blood vessels, some tumour cells and basal keratinocytes (data not shown).

### Lymphatic vessel density

Samples were analysed using a Nikon E-400 microscope using a × 40 objective. A ‘lymphatic’ was identified as a structure with LYVE-1-positive staining. The numbers of lymphatics found within a tumour were counted by eye and images recorded with a digital camera (coolpix 995; Nikon). A composite image was generated from the images and used to calculate the tumour and tissue area so that internal and external lymphatic densities could be calculated, respectively. All vessel counts were performed by one observer and lymphatic vessel density (LD) was calculated without knowledge of clinical data or prognostic outcome. LD was calculated as the number of lymphatic profiles per mm^2^ of section. Epitumoral LD was calculated as the LD within 350 *μ*m of the tumour edge (1 field of view). The median area examined was 2.2 mm^2^ epitumoral and 3.7 mm^2^ intratumoral in the MM samples. The median areas in the BCC and MCC samples were 2 and 5 mm^2^, respectively, intratumoral and 4.5 and 4.4 mm^2^ epitumoral; 12.3 mm^2^ of normal dermis was examined. Images were analysed using image analysis software NIH Image 1.62 to determine the area fraction of lymphatics, that is the area of the lymphatic per unit area of tissue.

Invasion of tumour cells into the lymphatics (lymphatic invasion) in a given tumour was considered to be present if tumour cells could be seen within any one LYVE-1 positive profile in the entire field. Invasion of blood capillaries and venules (vascular invasion) was considered to be present in a given tumour if tumour cells could be seen within any one LYVE-1 negative, PECAM-1-positive vessel within the tumour.

### Statistical analysis

The results are presented as a mean±s.e.m. Differences between lymphatic density and area fraction in tumour samples were analysed by Kruskal–Wallis test, a nonparametric one-way analysis of variance (ANOVA), followed by Dunn's *post hoc* test for multiple comparisons, and with Mann–Whitney *U*-test for single comparisons. To determine whether epi- and intratumoral LD or area fraction was different between metastatic and nonmetastatic, and *vice versa*, a two-way ANOVA and Bonferroni *post hoc* test was used. To determine significant differences of frequency of lymphatic and vascular invasion, Fisher's Exact test was used. *P*<0.05 was considered significant.

## RESULTS

### Epitumoral LD

**Figure 1:**
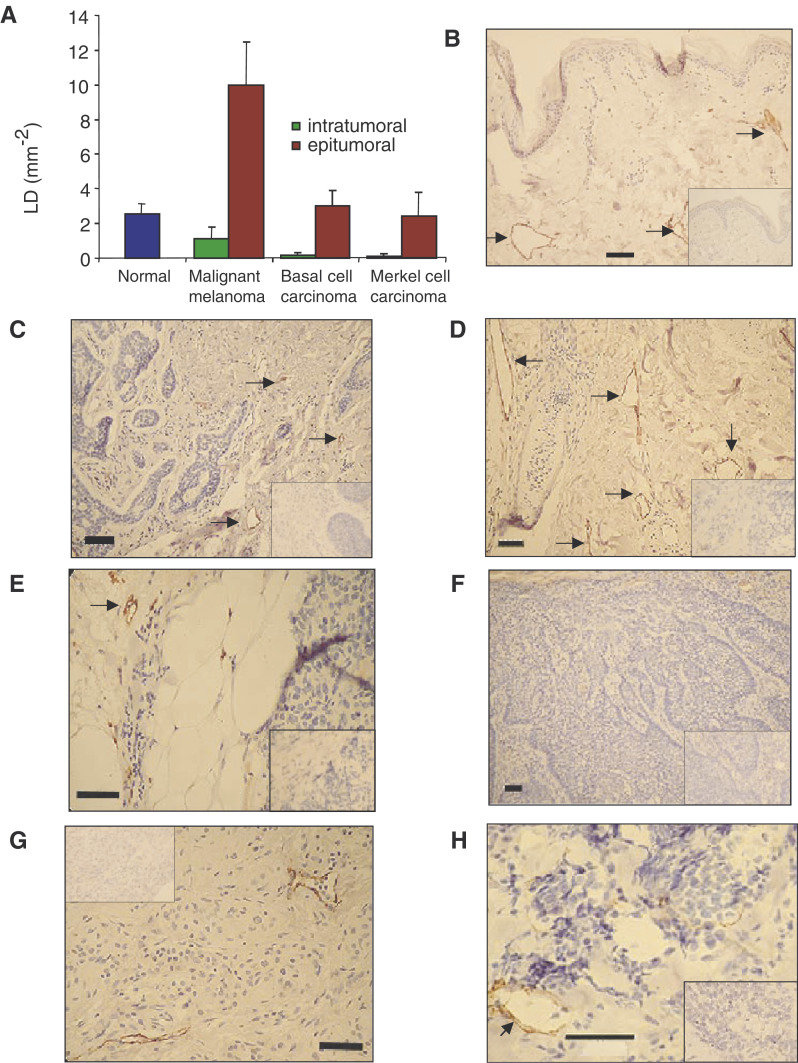
Lymphatic density in normal dermis, intra-tumoural LD and dermis immediately around three types of skin cancer. (**A**) Lymphatic density inside the tumour (green) was always lower than that outside the tumour (red). There was a significant increase in LD around the tumour in MM compared with normal dermis, BCC and MCC. See Results for values and detailed statistical analysis. Lymphatic capillaries in dermis stained with LYVE-1 antibody (arrows) in normal (**B**), BCC (**C**), MM (**D**) and MCC (**E**). Lymphatic vessels stained inside tumours in BCC (**F**), melanoma (**G**) and MCC (**H**). Inset is negative control staining. Bar: (**B–G)**, 50 *μ*m; (**H**), 100 *μ*m.

One-way ANOVA revealed significant differences in epitumoral LD (LD in the 350 *μ*m border around the three types of tumour) and the control dermis (*P*<0.0001, Figure 1

). Epitumoral LD around MMs (10.0±2.5 mm^−2^, *n*=21) was approximately four times higher than LD in the control dermis (2.4±0.9 mm^−2^, *n*=11, *P*<0.01 Dunn's multiple comparison test) and >3 times higher than epitumoral LD around BCC (3.0±0.9 mm^−2^, *n*=6, *P*<0.05 Dunn's test) or MCC(2.4±1.4 mm^−2^, *n*=6 , *P*<0.05 Dunn's test). The epitumoral LD around BCC and MCC were not significantly different from each other or from control LD (*P*>0.05, Dunn's test).

### Intratumoral LD

With regard to LD *inside* the tumours, one-way ANOVA again revealed significant differences in intratumoral LD among the three types of tumour and control dermis (*P*<0.01, Kruskal–Wallis test) (Figure 1). The intratumoral LD in the MMs (1.1±0.7 mm^−2^) was approximately eight times higher than intratumoral LD in BCC (0.14±0.19 mm^−2^) or MCC (0.12±0.16 mm^−2^), but not significantly lower than LD in the control dermis (2.4±0.9 mm^−2^). Intratumoral LD inside the BCC and MCC were both greatly reduced compared with the normal dermis (*P*<0.01, Dunn's *post hoc* test).

### Epi- *vs* intratumoral LD

**Figure 2:**
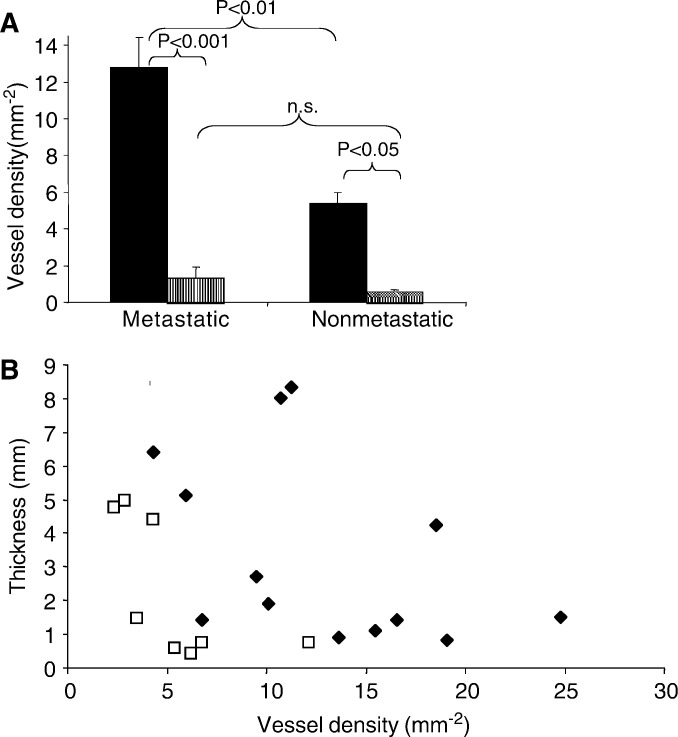
Relation between LD and metastasis in MM. (**A**) Lymphatic density outside (solid bars, mean±s.e.m.) and inside (stippled bars) MM classified according to whether the tumour had subsequently metastasised (*P*<0.001, two-way ANOVA). *Post hoc* tests showed significant difference between metastatic and nonmetastatic epitumoral LD (*P*<0.01, Mann–Whitney *U*), and between epi- and intratumoral LD for both nonmetastatic (*P*<0.05) and metastatic (*P*<0.001, Bonferroni), but not between metastatic and nonmetastatic intratumoral LD. (**B**) Plot of LD against thickness for nonmetastatic (open squares) and metastatic (filled diamonds) melanoma. There was no significant correlation for the pooled group (*r*=−0.2, *P*>0.1).

Two-way ANOVA showed that intratumoral LD was significantly lower for both metastatic and nonmetastatic tumours (*P*<0.01, metastatic, *P*<0.05 nonmetastatic, Figure 2A

) than epitumoral LD, but there was no difference between epi- and intratumoral LD of the tumours in BCC or MCC (*P*>0.05). As above, this was also true for the lymphatic area fraction in metastatic MM (*P*<0.001), but not in any other tumour type.

### LD of metastasising versus nonmetastasising MM

Epitumoral LD around the MMs was more than twice as great in patients who subsequently developed metastases (LD=12.8±1.6 mm^−2^, *n*=13, Figure 2A), than in those who had not yet developed metastasis at the time of writing (LD=5.4±1.1 mm^−2^, *n*=8) (*P*<0.01, Mann–Whitney *U*-test, and *P*<0.001 two-way ANOVA, Figure 2A). Although the intratumoral LD was likewise more than twice as great in the metastatic cases (LD=1.4±0.6 mm^−2^ as in the currently nonmetastatic cases (0.6±0.3 mm^−2^), this was not statistically significant. Although the thickness of an MM is prognostic in general, there was no relationship between thickness and LD in these patients (*r*=−0.26, *P*>0.1, Spearman rank correlation coefficient) (Figure 2B).

### Lymphatic area fraction

Lymphatic area fraction, calculated as the total intratumoral lymphatic area as a fraction of the entire area, was also increased in melanoma compared with normal, BCC and MCC and greater in metastatic melanoma than in nonmetastatic melanoma (see Table 1

**Table 1:** Lymphatic densities and area fractions in normal, BCC, MCC and MM

	**Lymphatic density (mm^−2^)**		**Lymphatic area fraction (%)**		
**Mean±s.e.m.**	**2.5±0.5**		**0.119±0.003^b^**		**N**
**Normal**	**Intratumoral**	**Epitumoral**	**Intratumoral**	**Epitumoral**	**11**
BCC	0.14±0.19^a^,^b^	3.0±0.9^b^,^c^	0.006±0.004^a^,^b^	0.123±0.021^a^	6
MCC	0.12±0.16^a^,^b^	2.4±1.4^b^,^c^	0.018±0.015^a^,^b^	0.158±0.089^a^	6
*MM*	1.1±0.7	10.0±2.5^c^,^d^	0.035±0.018^a^	0.760±0.304^d^	21
Nonmetastatic MM	0.64±0.29	5.4±1.1^c^	0.021±0.011	0.399±0.044	8
Metastatic MM	1.4±0.6	12.8±1.6^c^,^d^,^e^	0.043±0.013	0.982±0.244^d^,^c^,^e^	13

a Smaller than normal.

b Smaller than MM.

c Greater than intratumoral.

d Greater than normal.

e Greater than nonmetastatic.

). These results are similar to the LD results. There were no significant differences between the average size of the lymphatics (lymphatic area divided by the LD) between any of the tumours and normal tissue outside the tumours (*P*=0.46, one-way ANOVA, all mean±s.e.m. in mm^2^, 0.082±0.044 MM, 0.076±0.044 metastatic MM, 0.091±0.046 nonmetastatic MM; 0.055±0.039 BCC, 0.065±0.021 MCC, 0.045±0.005 normal).

### Vascular and lymphatic invasion

**Figure 3:**
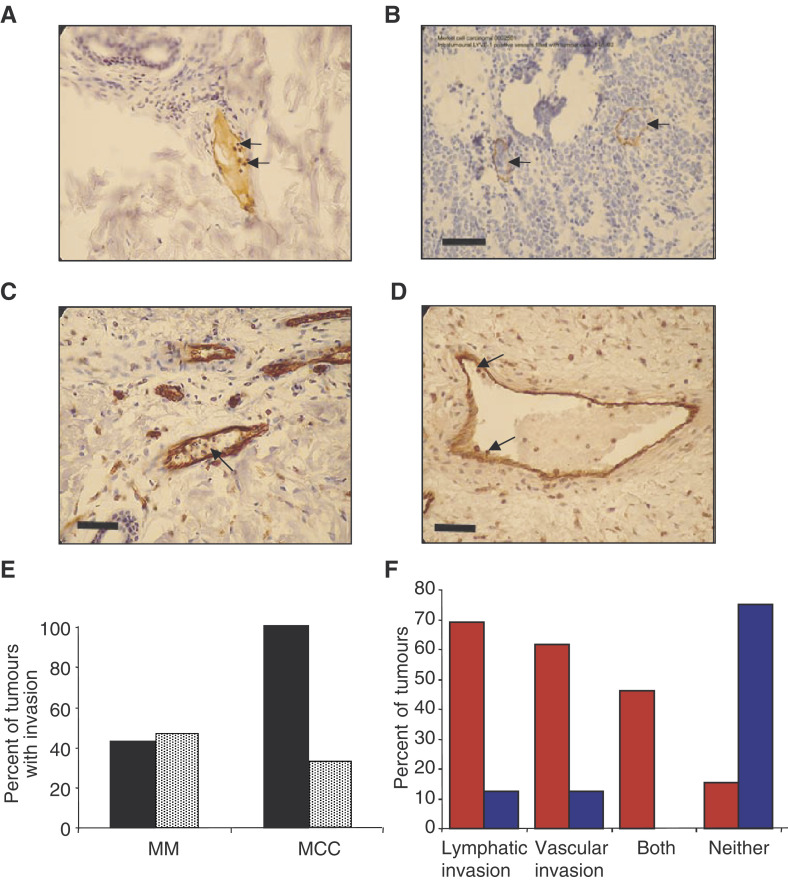
Lymphatic and vascular invasion in MM. Tumour cells were seen inside lymphatic capillaries (positive for LYVE-1) in both MM (**A**) and MCC (**B**). Vascular invasion (tumour cells inside PECAM positive, LYVE-1 negative vessels) was also seen in MM (**C**) and MCC (**D**). (**E**) Frequency of vascular (black) and lymphatic (stippled) invasion in MM and MCC. Although lymphatic and vascular invasion were equally common in MM, vascular invasion was significantly more common than lymphatic invasion in MCC *P*<0.05, Fisher's exact test. (**F**) Invasion frequency observed in metastatic (red) and nonmetastatic melanomas (blue). Lymphatic invasion alone, vascular invasion alone and both lymphatic and vascular invasion were significantly more common in metastatic than nonmetastatic melanomas. Combined vascular and lymphatic invasion was a particularly strong prognostic sign for metastasis (although not significantly different from either alone). Invasion of neither lymphatic nor vascular microvessels indicated a favourable prognosis, that is, no metastasis. Bar (**A, C, D**), 50 *μ*m; (**B**), 100 *μ*m.

Identification of the lymphatics in the tumour enabled vascular invasion to be distinguished from lymphatic invasion. In both the MM and MCC, vascular and lymphatic invasion were seen (Figure 3A–D

). In the six MCC, vascular invasion was more common (100% of tumours) than lymphatic invasion (33% of tumours, *P*<0.01, Fisher's Exact test). By contrast, there was no difference between lymphatic (47.2%) and vascular invasion (42.9%) in the melanoma samples (*P*=1.0, Fisher's Exact test) (Figure 3E). The combination of both lymphatic and vascular invasion in the same specimen was seen frequently in the metastasising melanomas (46%), but the combination did not occur in any of the nonmetastatic tumours (*P*<0.05 *χ*^2^ test). Therefore, although not all metastatic melanomas showed lymphatic invasion (Figure 3F), all the melanomas that showed both lymphatic and vascular invasion were metastatic. In the four metastatic melanomas without detectable lymphatic invasion, the LD (11.3±2.1 mm^−2^, *n*=4) was significantly greater than LD in those that had not metastasised (5.4±1.1 mm^−2^, *n*=11; *P*<0.05, state test used). There was no significant difference between the lymphatic densities of metastasised melanomas with (1.5±0.83 mm^−2^ intratumoral 13.5±2.21 mm^−2^ epitumoral) or without lymphatic invasion (1.15±0.42 mm^−2^ intratumoral 11.25±2.11 mm^−2^ epitumoral).

### Tumour thickness

There were no differences in the lymphatic densities between thin, medium or thick melanomas either inside or on the outside of the tumour (see Table 2

**Table 2:** Lymphatic densities of thick, intermediate and thin tumours

**Thickness (mm)**	**Intratumoral LD (mm^−2^)**	**Epitumoral LD (mm^−2^)**
<1	1.13±0.29	10.51±2.20
1–4	2.09±1.00	12.36±2.69
>4	0.18±0.07	7.51±1.97

), and in this sample the prognostic value of thickness was negligible (metastatic 3.36±0. 76 mm, nonmetastatic 2.28±0.72 mm, *P*=0.3, *t* test)

### Site and ulceration

There were no differences in the epitumoral lymphatic vessel densities between melanomas with either lymph node metastasis or metastasis to other sites (lymph node 11.1±1. 2 mm^−2^, other 16.6±4.36 mm^−2^, *P*=0.2 Mann–Whitney *U*-test), or between melanomas with or without ulceration (ulcerated 11.9±3.6 mm^−2^, nonulcerated 13.1±2.4 mm^−2^, *P*=0.92 Mann–Whitney *U*-test). Of the metastatic melanoma samples, 69% presented with lymph node involvement, 61.5% were nonulcerated, 23.1% were ulcerated and the status of 15.4% was unknown.

### Lymphatic endothelial proliferation and lymphangiogenic growth factors

Serial sections from all samples were taken and stained for proliferating lymphatic endothelial cells using successive LYVE-1 and Ki-67 staining. No staining was seen in any lymphatics. There was no clear evidence supporting the presence of proliferating lymphatics either within or around the melanomas, despite clear staining of the tumour tissue. VEGF-C and VEGF-D immunostaining was apparent in melanoma samples, but there were no qualitative differences in the intensity of staining for VEGF-C or VEGF-D in metastatic versus nonmetastatic melanoma samples.

## DISCUSSION

The mechanism through which MM spreads to the lymph nodes is key to understanding and preventing metastasis. Previous work in animal models has shown that overexpression of the lymphatic growth factor VEGF-C in breast cancer cells or beta cell tumours of the pancreas stimulates metastasis (Skobe *et al*, 2001b; Mandriota *et al*, 2001). The evidence is conflicting, however, on the role of lymphatics in human MM metastasis (Clarijs *et al*, 2001). Human MM *in situ* expresses VEGF-C (Salven *et al*, 1998), but direct evidence for lymphangiogenesis into the melanomas is lacking. Recently, however, overexpression of VEGF-C had been shown to stimulate lymphangiogenesis in xenotransplanted human melanomas (Skobe *et al*, 2001a), and in the chick chorioallantoic membrane, and this occurs through activation of VEGF receptor 3 (flt-4) in lymphatic endothelial cells (Papoutsi *et al*, 2000). Vascular endothelial growth factor **D**, another lymphangiogenic growth factor, has also been shown to be upregulated in melanoma and other tumours (Achen *et al*, 2001) (Yokoyama *et al*, 2003). Vascular endothelial growth factor C and -D staining were apparent in these samples, but there were no qualitative differences in the intensity of staining for VEGF-C or VEGF-D in metastatic *vs* nonmetastatic melanoma samples, as has been reported in other small-scale studies (Dadras *et al*, 2003). This is consistent with VEGF-C and -D not showing prognostic significance in melanoma compared to other tumours such as oral, breast or ovarian carcinoma (Kishimoto *et al*, 2003; Nakamura *et al*, 2003a, 2003b; Yokoyama *et al*, 2003).

There are a number of possible interpretations for the new findings presented in this study. The intratumoral LD in the MM was lower than LD in control dermis, but greater than in BCC or MCC. This might be due in principle either to the incorporation of some, but not all pre-existing lymphatics as the tumour invades the surrounding tissue, or due to the stimulation of vessel growth in an initially alymphatic tumour mass. Intratumoral LD was reduced almost to zero in BCC (a noninvasive, nonmetastasising tumour) and MCC (an invasive, distantly metastasising tumour). The intratumoral LD in the MM is closer to normal. Therefore, either some lymphatics must have been co-opted from normal tissue – a process that clearly does not happen in MCC or BCC, or lymphangiogenesis into the tumour must have occurred. The increase in epitumoral LD could be due to the tumour growing as a discrete entity and compacting the surrounding areas (hence increasing LD), or due to lymphangiogenesis, but again a comparison of MM with MCC and BCC supports the latter mechanism in MM. Another possibility – that MMs always grow in areas of skin containing a high LD – is not borne out by many decades of research into the relation between the site of the melanoma and the incidence of metastasis. There is no evidence of a relationship between the incidence of metastasis and sites with high LD (e.g. melanoma on the breast). The most likely explanation, therefore, for increased epi and intratumoral LD is that this particular tumour provides a particularly powerful lymphangiogenic stimulus.

Interestingly, there was a significantly greater LD around the melanomas than inside them, but the average size (cross-sectional area) of the lymphatics was not different. Since interstitial pressure is significantly higher inside the tumour than outside (Curti *et al*, 1993), and tumour vasculature is relatively leaky to fluid and solutes (Yuan *et al*, 1996), this would suggest that the majority of fluid cleared from the tumour goes through epitumoral lymphatics rather than intratumoral ones. This supports experiments that have shown a flow of fluid from the centre to the periphery of tumours, which may interfere with drug delivery to these tumours (Baxter and Jain, 1989). These results, however, do not provide evidence for or against the hypothesis that lymphatics inside tumours are not functional (Padera *et al*, 2002).

In this small study, there was no evidence of lymphatic proliferation at the time of excision. Therefore, active lymphangiogenesis could not be detected immunohistochemically. This neither confirms nor denies that lymphangiogenesis has occurred since the increase in LD must have occurred while the tumour was growing prior to excision and the newly formed tumour-stimulated lymphatics may be in a mature rather than in a proliferative state.

There has recently been one report that LD may be decreased in patients with metastasis, in contrast to the results shown here (Straume *et al*, 2003) and a second, very recently published, which supports the data described here (Dadras *et al*, 2003). The major difference between the current study and that of both Straume *et al* and Dadras *et al* was that we have determined the absolute LD around the melanoma by counting all the vessels, whereas both the other studies determined only the LD of the “hot spots” of lymphatics surrounding the melanoma. Straume *et al* found that the LD of the “hot spots” was reduced, and Dadras *et al* found that it was increased in samples from patients that developed metastasis. The difficulty with these findings is the subjective nature of the “hot spot”. A "hot spot" is an area of particularly high density of blood vessels as defined by Weidner *et al* (1991). Since this description, a few studies have used "hot spots" to quantitate lymphatic as well as vascular microvessel density in prevalent cancers other than melanoma (Birner *et al*, 2001; Maula *et al*, 2003). Hot spots, however, are representative of localised "biologically important" areas. This is more applicable to the growth of microvascular capillaries in response to the local release of growth factors stimulated by localised changes in oxygen tension. In addition, several reviews have outlined the difficulties with this technique, namely the subjective nature of the method; there is no defined cutoff point, and the influence of the measured area. Each of these variables leads to discrepancies between studies (Fox *et al*, 1995; Vermeulen *et al*, 2002). Since lymphatic growth does not appear to be regulated by hypoxia, there is no reason to assume that a functional increase in lymphatics will occur in "hot spots". Therefore, absolute LD may be a more appropriate and easily standardised measurement.

### Prognostic information

Tumour thickness is currently the most reliable predictor of recurrence for primary cutaneous melanoma and the single most important prognostic factor (Stadelmann *et al*, 1998). There is currently no consensus on the frequency of follow-up or recommendations for surveillance testing for all patients with melanoma, since at present there is no effective method to identify the small subgroup of patients with thin but aggressive MM. It would be helpful, therefore, to find a prognostic indicator to detect the high-risk patients in the group of “thin melanoma” (29% of the cases in this study). Since metastatic spread correlated with LD, even in thin melanomas (Figure 2), these results indicate that patients whose biopsy has a high LYVE-1 count could be included in a more intensive follow-up schedule, or considered for adjuvant treatment, even if the MM is thin. Furthermore, it may be possible, using LYVE-1 immunohistochemistry, to identify which of the thicker melanomas are not going to metastasise.

### Prognostic index

**Figure 4:**
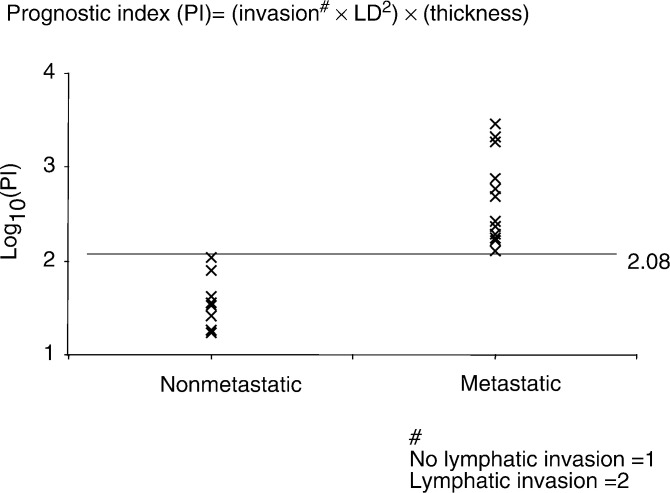
Prognostic index PI for MM plotted on a logarithmic scale. PI is the product of LD squared, thickness and a lymphatic invasion factor (1 for no invasion, 2 for lymphatic invasion). The inclusion of vascular invasion made no significant difference to the separation of the results from the two groups. All nonmetastatic tumours had a PI of <119 (log. value 2.08). All metastatic tumours were above this value.

Although thickness is a well-known prognostic factor, there are many cases of thin melanomas becoming metastatic, and many more of thick ones never metastasising. It would be useful, therefore, to increase the efficiency of prognosis by using LD and lymphatic invasion as prognostic factors. In this small study, LD correlated more closely with MM metastasis than did thickness. In order to determine whether, in this limited set of melanomas, there was any way of combining information on thickness, LD and lymphatic invasion, we calculated an index using all three terms, but weighted for LD rather than thickness. A prognostic index (PI) was calculated as the product of the LD squared (to weight the LD, since it appeared a better prognostic indicator than either lymphatic invasion or thickness), the thickness and a weighting number for lymphatic invasion (2 if present in a single LYVE-positive profile of the biopsy, 1 if not). Figure 4

shows PI for the each MM studied, grouped according to whether the MM had subsequently metastasised. In this, relatively small sample, PI discriminated between those tumours that have subsequently metastasised and those that have not done so after at least 6 years. The highest PI in nonmetastatic MM was 110 and the lowest PI in metastasising MM was 128, a 16% discrimination (see Figure 4). On the basis of the present, admittedly limited results, a putative prognostic index can be calculated that appears in this limited sample to be deterministic, thus PI appears to merit a larger multi-centre study. Further work is required to refine this PI, to further evaluate the lack of correlation between thickness and metastasis seen in this small study and to evaluate the incidence of false-negative and false-positive predictions.

In summary, we show here that the LD in the dermis surrounding MM is a good predictor of metastasis. The increased LD is probably brought about by lymphangiogenesis, which in turn supports the dissemination of cancer cells into the lymphatic system. A larger scale study of LD around MMs might be of value to determine the relative risk of metastasis with increased LD.
